# Systemic Suppression of the Shoot Metabolism upon Rice Root Nematode Infection

**DOI:** 10.1371/journal.pone.0106858

**Published:** 2014-09-12

**Authors:** Tina Kyndt, Simon Denil, Lander Bauters, Wim Van Criekinge, Tim De Meyer

**Affiliations:** 1 Department of Molecular Biotechnology, Ghent University, Ghent, Belgium; 2 Department of Mathematical Modelling, Statistics and Bioinformatics, Ghent University, Ghent, Belgium; 3 NXTGNT, Ghent University Hospital, Ghent, Belgium; University of Nebraska-Lincoln, United States of America

## Abstract

*Hirschmanniella oryzae* is the most common plant-parasitic nematode in flooded rice cultivation systems. These migratory animals penetrate the plant roots and feed on the root cells, creating large cavities, extensive root necrosis and rotting. The objective of this study was to investigate the systemic response of the rice plant upon root infection by this nematode. RNA sequencing was applied on the above-ground parts of the rice plants at 3 and 7 days post inoculation. The data revealed significant modifications in the primary metabolism of the plant shoot, with a general suppression of for instance chlorophyll biosynthesis, the brassinosteroid pathway, and amino acid production. In the secondary metabolism, we detected a repression of the isoprenoid and shikimate pathways. These molecular changes can have dramatic consequences for the growth and yield of the rice plants, and could potentially change their susceptibility to above-ground pathogens and pests.

## Introduction

Rice (*Oryza sativa L*.) is one of the most important staple foods in the world, feeding more than 50% of the human population. One of the most damaging pathogens, with major impact on rice yield, is the migratory endoparasitic nematode *Hirschmanniella oryzae*. *Hirschmanniella oryzae* is present in the soil and irrigation water of the majority of rice-growing areas, mainly where rice is grown under submerged conditions [1], [2]. These nematodes penetrate plant roots and move through the cortex of the root, producing cavities and channels and eventually necrosis. Roots invaded by *H. oryzae* exhibit discoloration, deterioration and rotting [1], while heavily infested plants also show above-ground symptoms like stunting and up to 60% reduced tillering [1], [2].

In comparison with the existing knowledge on the infection process of dicots by sedentary endoparasitic nematodes [3], far less is known about the interaction between monocot plants and nematodes or plant interactions with migratory endoparasitic nematode species. Plants generally respond to nematode infection by differential expression of genes involved in stress and defense responses, cell wall alteration, metabolism and nutrient allocation, and signal transduction [3]. Our previous transcriptome analysis on the local response of rice roots upon infection with the *H. oryzae* revealed that at 3 days after inoculation the infected roots accumulated mRNA of many biotic stress-related genes, oxidative stress and cell death pathways [4]. Concurrently the normal primary metabolism of the rice roots appeared impaired. Particularly the jasmonate and ethylene pathways, which are known to be involved in wound responses [5], were strongly induced upon migratory endoparasitic nematode infection in rice roots, although some suppression of specific defense responses was observed at later time points [4]. Next to the accumulating evidence on gene expression and metabolite changes in locally infected tissues, research has also provided evidence on the nematode-induced systemic changes in pathogenesis-related protein levels [6], [7], [8], primary metabolites [9], and hormone-related defense pathways [10]. Below-ground feeding organisms such as insects, nematodes, root pathogens, and ectomycorrhizal fungi are known to influence the concentration of above-ground plant defense compounds including terpenoids, glucosinolates, and phenolics [11], [12], [13], [14], leading to an impact on the susceptibility of the above-ground tissues to subsequent pathogen attack (reviewed in [15]). However, the molecular mechanisms and associated genes driving such a systemic response remain barely investigated.

Therefore, to gain deeper insight into the systemic transcriptional changes in rice after *H. oryzae* infection we have performed mRNA-Seq on the shoots of rice root nematode infected plants. The observations were independently validated using qRT-PCR and biochemical analyses. This research reveals significant modifications in the metabolism of the plant, with a general suppression of chlorophyll biosynthesis and primary metabolic processes involved in plant growth.

## Materials and Methods

### Infection

*Oryza sativa* cv. ‘Nipponbare’ (GSOR-100, USDA) was germinated on wet filter paper for 6 days at 30°C and then transferred to SAP-substrate (Sand and Absorbent Polymer; [16]) and further grown at 26°C under a 16 h/8 h light-regime. *Hirschmanniella oryzae* infected rice roots were collected in rice fields in Myanmar and sent to Ghent University by Zin Thu Zar Maung (Plant Protection Division, Yangon, Myanmar). This sampling was done at Padan Village, Hlaingtharyar Region in Yangon Division, Myanmar. As this did not involve endangered or protected species, no specific permissions were required. The nematode-infected rice roots were imported in Belgium, issued by permit BE/LOA/2012/033. All further experiments were done at Ghent University, authorized by biosafety permit T38-0428, activity number 13.

The nematodes were extracted using the Baermann funnel method [17]. Eighteen-day-old rice plants were inoculated with a mixed population of *H. oryzae* at the rate of 400 per plant, or mock-inoculated with water. This was done by inserting a 1 ml pipette tip just adjacent to the plant root system and releasing the nematode suspension (dissolved in water). One day after inoculation the plants were transferred to a hydroponic culturing system with Hoagland solution [16] to synchronize the infection process. Shoots of inoculated and non-inoculated plants were collected 3 and 7 days post inoculation (dpi). At each time point two independent biological replicates, each containing a pool of 6 plants, were collected for RNA-Sequencing. A third independent biological replicate of both inoculated and non-inoculated plants, again each containing a pool of 6 different plants, was used for qRT-PCR validation.

### RNA extraction, library preparation and sequencing

RNA was extracted using the Qiagen RNeasy Plant Mini Kit (Qiagen), with an additional sonication step after addition of buffer RLT. RNA integrity was checked using the Agilent BioAnalyzer 2100 (Agilent). Approximately 2 µg of total RNA was used for mRNA-Seq library construction according to the manufacturer’s recommendations (Illumina).

We used the multiplexing sequencing adapters provided in the Multiplexing Sample Preparation Oligo Kit (Illumina). Size selection of the library was performed on a 2% agarose gel (Low Range Ultra Agarose, Biorad 161-3107). The denatured library was diluted to a final concentration of 6 pM and loaded on a paired-end read flow cell (TruSeq v5 kit, Illumina). To minimize lane effects the samples were multiplexed. Each sample was sequenced in duplicate in 2 different lanes (4 lanes total with 8 MID tags per lane). After cluster generation, the multiplexed library was sequenced on an Illumina Genome Analyzer IIx (36 cycles, paired end).

### Mapping reads to genome data and annotated transcripts

Reads were mapped to the *Oryza sativa* subsp. *japonica* reference genome (build MSU7.0) in two phases using TopHat version 1.3.1 [18] and Cufflinks, version 1.0.3 [19]. The workflow and settings used in the data analysis have previously been described [4].

### Calculation, normalization and profiling of gene expression

Expression was quantified per sample and per annotated or unannotated transcript as the sum of all reads mapped to the respective gene exons with a 16 base pair tolerance on either side to compensate for potential errors in the gene annotation. Expression profiles were assessed using the R-package “baySeq”, version 1.5.1. [20]. To compensate for artificial differences in read distributions, the original library sizes were multiplied by additional normalisation factors calculated using the Trimmed Median of M-values method described in [21] with standard settings as implemented in the edgeR package (version 2.0.3). For statistical significance an FDR cut-off of 0.05 was considered for the ‘general response’ analysis, where the time point after inoculation was not taken into account. For the analysis per time point (3 and 7 dpi), considering that only 2 biological replicates (although pools) per treatment were analyzed for these tissues, the statistical analysis was performed with less stringent conditions (FDR<0.1).

For all further analyses the expression level of each transcript for each condition was estimated as the fold change (FC) of mapped reads relative to the controls. The FC was calculated as follows: reads were normalized as described earlier and averaged over the biological replicates. Before calculating the base 2 log of the ratio of these averages, the number of reads was increased by 1 in each group (to avoid 0-values).

### Gene ontology and enrichment analyses

Gene ontology (GO) analysis and GO enrichment were performed using agriGO [22]. Parametric Analysis of Gene Set Enrichment (PAGE) [23], based on differential gene expression levels (log_2_FC), was executed. Benjamin and Hochberg false discovery rate analysis (FDR) was performed to adjust the PAGE P-values.

In addition, MapMan [24] was used to visualize the expression of genes onto metabolic pathways and the WSR-test (with Benjamin and Hochberg correction) was used to test the statistical significance of differential expression of these pathways.

### Analysis of Novel Transcriptionally Active Regions (nTARs)

The Cufflinks program generates a GTF file including all transcripts annotated in MSU7.0 and putative novel transcripts derived from the data. BLASTx searches were performed against Swiss-Prot and trEMBL and all predicted rice proteins (http://rice.plantbiology.msu.edu/). Homologues of the nTARs in rice ESTs (downloaded from NCBI on February 1, 2014) were searched by BLASTn. For all analyses a bitscore cut-off of 50 was used.

### Validation of mRNA-Seq by qRT-PCR

Based on potential functional importance, a subset of genes was selected for validation in an independent biological sample (pool of 6 plants) by qRT-PCR, at 3 dpi. Locus numbers of these transcripts and primer sequences are presented in Table 1. PCRs were performed under the following conditions: 10 min at 95°C and 45 cycles of [25 s at 95°C, 60 s at 58°C and 20 s at 72°C]. After the PCR reaction, a melting curve was generated by gradually increasing the temperature to 95°C to test the length as an indication of PCR specificity. Normalization of the expression level of the target genes was done using two reference genes (LOC_Os03g27010, [25]; LOC_Os07g02340.1, [26]). Data was analysed as previously described [4].

**Table 1:** qRT-PCR validation of a selection of genes.

				Relative geneexpression level(infected versuscontrol tissue)		
Locus numer	Annotation	primerF	primerR	RNA-Seq	qRT-PCR	Primer efficiency (%)
LOC_Os03g16010	BRASSINOSTEROID INSENSITIVE 1-associated receptor kinase 1 precursor	TTCAGGGTGGAGCTATGTGG	TTTATAAACCGTCCCGAAGC	0.27	0.85	74
LOC_Os02g17640	isochorismatase	TCTTTGATGCTGTAGCACTGG	AAGGTTGGTGTTTCCACTCC	0.29	0.36	74
LOC_Os01g40094	protein phosphatase 2C, putative,expressed	GGGAGGATGAATATGCAAGG	CTCGGGGACTGGAATTATCC	0.34	0.79	76
Loc_Os01g55870	chorismaat mutase	ATGGTAAGTTTGTGGCAGAGG	CCACCGTTTCATAGGTGAGG	0.35	0.53	73
LOC_Os03g14730	gibberellin receptor GID1L2, putative, expressed	GTGGAGGCACTCCAGAAAGC	CTCCTTGAACAGCTCCATCG	0.38	0.55	69
LOC_Os01g08450	ras-related protein,putative, expressed	GGAGACAAGTGCCAAGAACG	TCTTCTGGTTGACAGGTTGC	0.45	0.48	75
LOC_Os10g40570	flavin-containing monooxygenasefamily protein, putative, expressed	GGATGGCCTAAGAGATACGC	ATTGTTCAATTGGTGGATGG	0.49	0.37	65
LOC_Os03g04920	multidrug resistance- associated protein,putative, expressed	ACCATTGCACACCGTATCC	AGGACTTGTCCTCCAAGAGC	0.51	0.61	73
LOC_Os05g35290	Phenylalanine ammonia-lyase,ZB8, putative, expressed	CTCGTACCCGCTCTACCG	GACGAGCACCTTGTTGAGC	0.88	0.48	70
LOC_Os01g67030	auxin-responsive protein, putative,expressed	CCAACCTCACCTGCTTCG	CACGTACCCTCCCTTGTCC	1.84	0.51	70
LOC_Os04g41070	zinc finger, C3HC4 type domaincontaining protein, expressed	CTGGAGGCTCAGGACCAG	CAGAAGTGGCTCCTCCAAAC	1.91	1.06	71
LOC_Os09g31450	Polygalacturonase inhibitor 1precursor, putative, expressed	TCAACGTCAGCGACAACG	ACCTGTTGCGAGCGTAGC	2.00	1.23	69
LOC_Os04g18650	AP2 domain containingprotein, expressed	GAGAAGGTGGAGCTTGTGTACC	GTTGTTGTTGTTGCGGTAGC	2.38	1.36	75
LOC_Os02g33840	OsFBX52 - F-box domaincontaining protein,expressed	GAGTCCTTCGGGTGGATACC	CCTGAAGAACCCAGACATCC	2.56	0.71	65
LOC_Os03g58990	cupin domain containingprotein, expressed	ACGTATCGTCGCCGTACAAC	GACGGCGTCGGGTATCTC	3.29	1.29	72
LOC_Os11g42290	transferase family protein,putative, expressed	CGAGTACATCCAGTCGTTCG	CATCTCGTGGAACCTGAACC	3.95	1.35	69

The table shows the relative expression levels, obtained using mRNA-Seq or qRT-PCR on an independent sample, comparing systemic tissue of *H. oryzae* infected plants with uninfected plants at time point 3 dpi.

### Chlorophyll measurement

Chlorophyll measurements were performed in an independent experiment. Nematode collection, as well as growth conditions and inoculation of the plants were as described above. Shoots of inoculated and non-inoculated plants were harvested at 3 and 7 dpi. At each time point and for each treatment, 12 individual plants were used. At the day of sampling the length of shoot and root of each plant was measured. Then, shoots were harvested and pooled in 4 samples, each pool containing shoots of 3 individual plants. In each sample, chlorophyll content was determined after extraction in ice-cooled 100% Methanol and spectrophotometrical measurement of the absorbance (A) at 665.2 (chlorophyll a; Chl_a_) and 652 nm (chlorophyll b; Chl_b_). To calculate the concentration in mg/L following formulas were applied: Chl_a_ = 16.29 * (A_665.2_)–8.54 * (A_652_); Chl_b_ = 30.66 * (A_652_)–13.58 * (A_665.2_) [27]. After checking for normality and homoscedasticity of the data, statistical significance was evaluated using student’s t-test. This whole experiment was repeated in time, with similar results.

## Results

More than 95 million RNA fragments were acquired and made publicly available in the GEO database (GSE57707). The short reads were aligned against the reference genome sequence of cv. Nipponbare (MSU7.0) and in total 81% of the sequenced reads could be mapped (Table 2). The total number of sequenced bases was nearly 7 billion, representing 18-fold the rice genome size and about 50-fold coverage of the annotated transcriptome. The expression of a total of 89,246 different rice transcripts was detected in the analyzed tissues. Comparative gene expression profiling was performed by statistical analysis of differential gene expression levels, Gene Set Enrichment, and pathway mapping. In addition, a search was performed to detect novel transcriptionally active regions (nTARs) not annotated in rice genome assembly MSU7.0.

**Table 2:** Overview of the obtained sequencing data and mapping of these sequences onto the rice genome.

Sample	Total number of sequencedread pairs	Total number of mappedread pairs	Number of uniquelymapped read pairs	Number of unmapped readpairs
Shoots of uninfected plants,3 dpi, replicate 1	9407160	16793253	7475187	1931973
Shoots of uninfected plants,3 dpi, replicate 2	12402694	22365580	9863309	2539385
Shoots of uninfected plants,7 dpi, replicate 1	12338830	22597722	9838088	2500742
Shoots of uninfected plants,7 dpi, replicate 2	11957710	22739886	9557445	2400265
Total of shoot tissue ofuninfected plants	46106394	84496441	36734029	9372365
Shoots of Ho-infected plants,3 dpi, replicate 1	10819261	21665010	8489566	2329695
Shoots of Ho-infected plants,3 dpi, replicate 2	13166886	29099106	10981032	2185854
Shoots of Ho-infected plants,7 dpi, replicate 1	12601325	25281161	10705989	1895336
Shoots of Ho-infected plants,7 dpi, replicate 2	13099442	25818811	10737960	2361482
Total of shoot tissue ofinfected plants	38867653	80199078	32424981	6442672
**Overall Total**	**95793308**	**186360529**	**77648576**	**18144732**
sequenced bases perfragment (ie read pair)	72			
number of sequenced bases	6.90E+09			
length of rice genome(approximate)	374424240			
length of rice transcriptome(approximate)	139985298			
genome coverage	18			
transcriptome coverage	49			

### Systemic transcriptome changes upon rice root nematode infection

In a first analysis, the expression level in all sampled systemic tissues of infected plants (at both time points) was compared with the shoot tissues of healthy plants to look for consistent trends, regardless of the time point after inoculation.

At an FDR cut-off of 0.05, 52 annotated genes were found to be significantly differentially expressed in the shoots after root infection with *H. oryzae* (Table 3). Twenty one of these differentially expressed genes (DEGs) were significantly downregulated, while 31 showed higher expression in infected plant shoots. Among the downregulated genes for instance, we identified one encoding a RAS-related protein (Small monomeric GTPase), and a gene encoding 3-oxo-5-alpha-steroid 4-dehydrogenase, involved in brassinosteroid biosynthesis. Among the upregulated DEGs, 2 genes encoding AP2 domain containing proteins, and a cupin domain containing protein were found. Next to the annotated transcripts, 12 nTARs were found to be differentially expressed in shoots of inoculated vs. non-inoculated plants (Table 3).

**Table 3:** Differentially expressed genes (FDR<0.05), when comparing systemic tissue of *H. oryzae* infected plants with uninfected plants at both time points (3 and 7 dpi).

Gene_ID	Annotation	Log_2_FC (shoots of infectedversus uninfected plants)	FDR
RNSR_1.36318	No Description	–3.58	0.02
LOC_Os05g34270	inactive receptor kinase At1g27190precursor, putative, expressed	–2.00	0.01
LOC_Os12g29670	expressed protein	–1.93	0.03
LOC_Os01g62950	ras-related protein, putative, expressed	–1.85	0.02
LOC_Os06g05130	myristoyl-acyl carrier proteinthioesterase, chloroplast precursor,putative, expressed	–1.83	0.04
LOC_Os05g36290	actin, putative, expressed	–1.73	0.01
LOC_Os10g02350	transmembrane 9 superfamilymember, putative, expressed	–1.71	0.05
LOC_Os01g54940	dehydrogenase, putative, expressed	–1.69	0.01
LOC_Os06g07630	26S protease regulatory subunit6A, putative, expressed	–1.68	0.04
LOC_Os03g52040	OsSCP19 - Putative SerineCarboxypeptidase homologue, expressed	–1.65	0.04
LOC_Os02g36330	RING-H2 finger proteinATL1O, putative, expressed	–1.60	0.02
RNSR_1.55235	No Description	–1.55	0.03
LOC_Os04g48750	3-oxo-5-alpha-steroid4-dehydrogenase, putative, expressed	–1.51	0.05
RNSR_1.61315	No Description	–1.50	0.03
LOC_Os02g52010	phosphate-induced protein 1conserved region domaincontaining protein, expressed	–1.43	0.04
LOC_Os01g07950	OsGrx_S15.2 – glutaredoxinsubgroup II, expressed	–1.39	0.02
RNSR_1.28714	No Description	–1.35	0.01
LOC_Os02g38810	26S proteasome non-ATPaseregulatory subunit 6,putative, expressed	–1.34	0.04
LOC_Os04g56510	ABC1 family domain containingprotein, putative, expressed	–1.28	0.03
LOC_Os02g46839	expressed protein	–1.14	0.04
LOC_Os02g56900	thioredoxin family protein,putative, expressed	–1.09	0.02
LOC_Os04g33600	hydrolase, alpha/beta foldfamily protein, putative, expressed	–1.08	0.02
LOC_Os05g45040	OsFBX170 - F-box domaincontaining protein, expressed	–1.01	0.03
LOC_Os01g37700	expressed protein	–0.88	0.04
LOC_Os01g39310	SEY1, putative, expressed	–0.67	0.04
LOC_Os10g28580	CAF1 family ribonucleasecontaining protein, expressed	0.52	0.03
LOC_Os10g22300	resistance protein,putative, expressed	0.59	0.03
RNSR_1.47341	No Description	0.77	0.03
RNSR_1.30534	No Description	0.82	0.03
RNSR_1.4486	No Description	0.82	0.04
RNSR_1.42503	No Description	0.84	0.01
LOC_Os05g31420	GRAS family transcriptionfactor containing protein, expressed	0.88	0.04
RNSR_1.27029	No Description	0.95	0.01
LOC_Os06g45460	OsFBX202 - F-box domaincontaining protein, expressed	0.97	0.04
LOC_Os09g31450	polygalacturonase inhibitor 1precursor, putative, expressed	0.97	0.03
LOC_Os05g40570	expressed protein	0.99	0.04
LOC_Os01g67030	auxin-responsive protein,putative, expressed	1.02	0.02
LOC_Os03g48590	protein transport proteinSec61 subunit alpha,putative, expressed	1.03	0.05
LOC_Os02g33840	OsFBX52 - F-box domaincontaining protein, expressed	1.08	0.03
LOC_Os01g41140	THION18 - Plant thioninfamily protein precursor, expressed	1.14	0.01
LOC_Os04g41070	zinc finger, C3HC4 typedomain containing protein, expressed	1.16	0.02
LOC_Os12g09630	expressed protein	1.20	0.03
LOC_Os05g10300	expressed protein	1.25	0.03
LOC_Os08g04740	expressed protein	1.26	0.04
LOC_Os03g42259	hypervariable Bacillus group-specificprotein, putative, expressed	1.30	0.05
LOC_Os07g39450	expressed protein	1.32	0.04
LOC_Os05g08580	hypothetical protein	1.33	0.02
LOC_Os05g49010	AP2 domain containingprotein, expressed	1.35	0.05
RNSR_1.17134	No Description	1.38	0.01
LOC_Os10g23840	conserved hypothetical protein	1.58	0.02
LOC_Os11g42290	transferase family protein,putative, expressed	1.64	0.04
LOC_Os09g20470	hypothetical protein	1.71	0.02
LOC_Os03g58990	cupin domain containingprotein, expressed	1.75	0.03
LOC_Os04g02470	expressed protein	1.75	0.03
LOC_Os04g18650	AP2 domain containingprotein, expressed	1.87	0.01
LOC_Os12g10370	expressed protein	1.97	0.02
LOC_Os03g31280	expressed protein	1.98	0.02
LOC_Os08g02200	expressed protein	2.02	0.01
LOC_Os06g08270	expressed protein	2.03	0.05
LOC_Os02g32310	expressed protein	2.27	0.05
LOC_Os12g13540	hypothetical protein	2.36	0.03
RNSR_1.34363	No Description	2.46	0.03
RNSR_1.11099	No Description	2.54	0.04
LOC_Os12g27040	expressed protein	2.58	0.02

The table shows the rice locus number, the annotation (MSU7.0), the Log_2_ of the fold-change (FC) of infected versus uninfected shoot tissue and the FDR-value. Novel transcriptionally active regions received a gene ID starting with RNSR. FDR: false discovery rate. RNSR: Root Nematode Systemic Respons.

**Figure 1:**
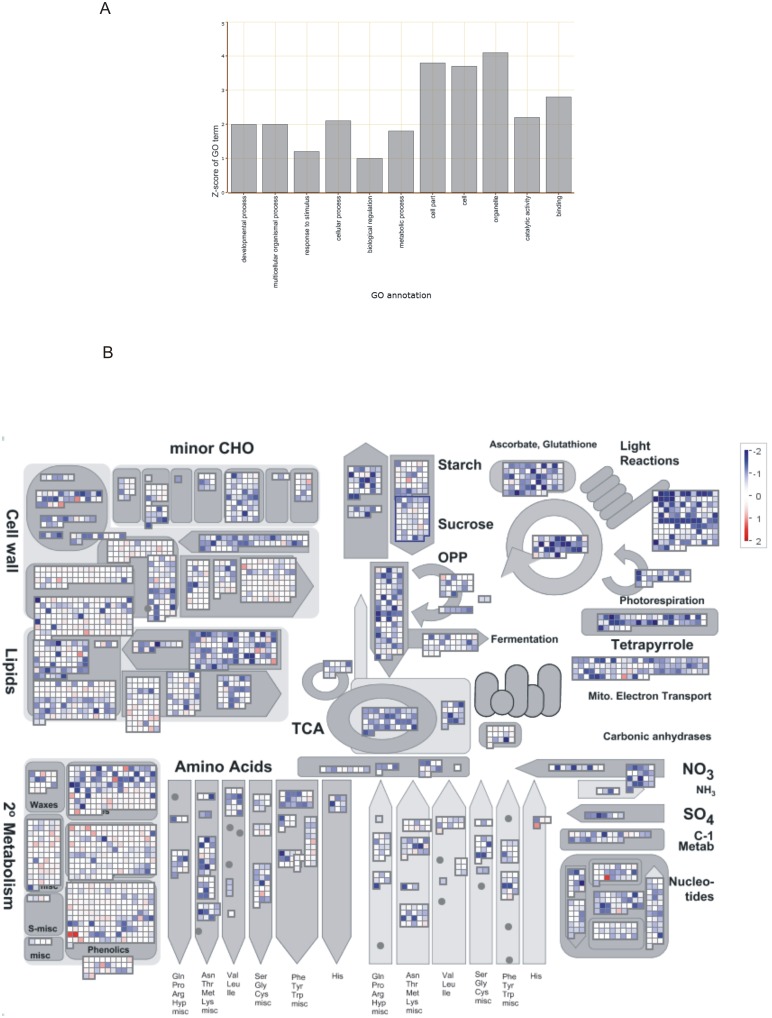
Visualization of transcriptome data of shoots of rice infected with rice root nematode *Hirschmanniella oryzae*. (A) Parametric Analysis of Gene Set Enrichment of the differentially expressed genes in the shoots of infected versus uninfected plants. The Z-scores of the significantly enriched secondary level GO terms are shown. (B) Mapman visualization of the expression profiles of genes involved in the general metabolism of the rice plant. The visualization shows the observed differential expression patterns, based on the Log_2_ fold changes of mRNA levels, in shoots of infected versus uninfected control plants. Red dots indicate that the gene is upregulated in infected plants versus the corresponding healthy control plants, while blue indicates downregulation.

Parametric Analysis of Gene Set Enrichment of the differentially expressed genes in the infected versus uninfected plants revealed that the repressed genes were enriched for the secondary GO-groups ‘developmental process’, ‘multicellular organismal process’, ‘cellular process’, ‘metabolic process’, ‘organelle’, ‘cell’, ‘cell part’, ‘catalytic activity’ and ‘binding’ (Fig. 1A). None of the enriched gene sets showed induction in systemic tissue of infected plants in comparison with control shoot tissue.

When focusing on the general plant metabolism (Fig. 1B) the WSR-test of Mapman found a significant suppression of many primary metabolic processes, like ‘light reaction’, ‘fatty acid synthesis and elongation’, ‘ATP synthesis’, ‘tetrapyrrole synthesis’, ‘nucleotide metabolism’, ‘amino acid synthesis’, ‘TCA’, ‘glycolysis’, and ‘cell wall precursor synthesis’. Not only the primary metabolism of the plants was impaired, we also detected a specific suppression of a few plant defense related pathways. For instance, the biosynthesis of chorismate through the shikimate pathway, was strongly suppressed in the systemic tissue. Additionally, a significant suppression of the ‘isoprenoids’ pathway was observed.

The impact of the observed transcriptome changes in the primary metabolic pathways was analyzed by measuring the length of roots and shoots of infected versus uninfected plants. At 3 and 7 dpi, plant root and shoot length was not different from the control plants (data not shown). Because ‘light reaction’ and ‘tetrapyrrole synthesis’ were among the gene sets with the strongest levels of suppression upon infection, the chlorophyll content was measured in the leaves of *H. oryzae*-infected and control plants at 3 and 7 dpi (Table 4). At 3 dpi, a decreasing trend in levels of both chlorophyll a and b was detected when comparing infected with uninfected rice plants. At 7 dpi, a significant reduction in chlorophyll a was detected (18%) in the infected plants, and although not significant, the infected plants also showed 10% reduction in chlorophyll b content in comparison with uninfected control plants.

**Table 4:** Chlorophyll a and chlorophyll b content of shoots of plants infected with rice root nematode *H. oryzae*, in comparison with control (uninfected) plants of the same age.

	Chlorofyll a	Chlorofyll b
**Shoots of uninfected plants, 3 dpi**	704.7±150.6	147.9±29.2
**Shoots of infected plants, 3 dpi**	646.0±91.4	121.2±19.5*
**Shoots of uninfected plants, 7 dpi**	809.0±76.3	150.6±15.0
**Shoots of infected plants, 7 dpi**	666.3±34.05**	135.2±14.4

Values are the average ± standard deviation of four pools (n = 4) of shoot material from three plants each. Statistically significant differences in comparison with the respective control (at the same time point) are indicated with asterisks.

**P<0.05,

*P<0.1.

### Systemic transcriptome changes at 3 days after rice root nematode infection

When considering the 3 dpi samples alone, only 2 biological replicates (although pools) per treatment were analyzed, and therefore the statistical analysis was performed with less stringent conditions (FDR<0.1). This resulted in 195 DEGs, with 169 repressed and 26 induced (Table S1). Next to the annotated transcripts, 25 nTARs were found to be differentially expressed in this tissue versus uninfected plant shoots (Table S1). Pathway mapping with MapMan showed a general suppression of the plant primary metabolism in the systemic tissues at 3 dpi (Figure S1).

Among the most intriguing and strongest downregulated DEGs were genes encoding (1) chalcone-flavonone isomerase (LOC_Os11g02440; Log_2_FC = –2.75), involved in flavonoid biosynthesis, (2) 3-beta hydroxysteroid dehydrogenase/isomerase family protein (LOC_Os03g23980; Log_2_FC = –2.27), involved in brassinosteroid biosynthesis, (3) the transcript encoding the brassinosteroid cell-surface leucine-rich repeat receptor-like kinase (LRR-RLK) BRASSINOSTEROID-INSENSITIVE 1 (BRI1, LOC_Os01g17250; Log_2_FC = –1.64), and (4) 2 other LRR-RLKs (LOC_Os02g09740, Log_2_FC = –2.24; LOC_Os06g47710, Log_2_FC = –2.84). LRR-RLKs regulate a wide variety of developmental and defense-related processes including cell proliferation, stem cell maintenance, hormone perception, host-specific as well as non-host-specific defense response, wounding response, and symbiosis [28]. Two other suppressed DEGs were encoding enzymes belonging to the shikimate pathway, chorismate mutase (Log_2_FC = –1.51) and isochorismatase (Log_2_FC = –1.78).

Among the induced DEGs, (1) a terpene synthase (LOC_Os12g30824; Log_2_FC = 1.10), necessary for the biosynthesis of ent-sandaracopimaradiene, a precursor of the diterpenoid phytoalexins oryzalexin A–F, (2) LOC_Os07g10240 (Log_2_FC = 2.05) encoding an anthocyanidin 3-O-glucosyltransferase involved in flavonoid (anthocyanin) biosynthesis, as well as many unannotated genes and 12 nTARs (Table S1).

To validate the gene expression levels as detected by RNA-seq, an independent validation was performed using qRT-PCR, comparing infected with uninfected plants at 3 dpi. In total, 14 out of the 16 tested gene expression profiles (87.5%) were confirmed (Table 1), in line with an expected FDR of 10%.

### Systemic transcriptome changes at 7 days after rice root nematode infection

For the 7 dpi data, the statistical analysis was again performed with less stringent conditions (FDR<0.1), resulting in 17 DEGs, with 11 repressed and 6 induced. Next to the annotated transcripts, 10 nTARs were found to be differentially expressed in this tissue versus uninfected plant shoots (table S2). Most induced DEGs are annotated as ‘expressed protein’ or ‘hypothetical protein’, except for a gene annotated as heat shock protein DnaJ (LOC_Os01g42190; Log_2_FC = 0.528), and LOC_Os04g18650 (Log_2_FC = 1.652), coding for a pathogenesis-related transcriptional factor, with an ethylene-responsive AP2 domain (EREBP34, TSRF1, belonging to class IIIb; [29]). The tomato homolog of this AP2 gene, TSRF1 has previously been shown to enhance the osmotic and drought tolerance of rice [30]. This locus was also slightly (but not significantly) induced at 3 dpi in the systemic leaf tissue. Among the most strongly repressed DEGs are (1) a MYB family transcription factor (LOC_Os08g06110; Log_2_FC = –2.799), (2) a ribonuclease T2 family domain containing protein (LOC_Os08g33710; Log_2_FC = –2.250), (3) a CTP synthase (LOC_Os05g49770; Log_2_FC = –2.149), (4) monodehydroascorbate reductase (LOC_Os08g44340; Log_2_FC = –1.734), and (5) lactate/malate dehydrogenase (LOC_Os08g33720; Log_2_FC = –1.407).

### Analysis of novel transcripts

A total number of 33,638 nTARs were detected in the analysed tissues (Table S3). A BLAST search against all ESTs from *O. sativa* resulted in 30,214 nTARs giving a significant hit (bitscore>50), while tBLASTx against all proteins of *O. sativa* resulted in significant hits for 19,735 of the nTARs, indicating that these nTARs are potential paralogues of known rice transcripts (Table S3). A Singular Enrichment analysis (FDR<0.05) of these rice transcripts revealed four enriched GO-terms of the category ‘Molecular function’: kinase activity, transferase activity, transferase activity - transferring phosphorus-containing groups and catalytic activity. A SwissProt search was done, and this was successful for 6,271 transcripts (Table S3), revealing for instance nTARs showing resemblance to protein domains annotated as xylanase inhibitor, MATE efflux family protein, histone-lysine N-methyltransferase, ribonuclease H protein, sugar transport protein, and ethylene-responsive transcription factor ABI4. A BLAST search against Trembl resulted in 16,134 hits, mainly to uncharacterized proteins from *O. sativa*.

## Discussion

Gene expression analyses investigating the plant response upon nematode attack have mainly targeted the local reaction of the plant [4], [25], [31], [32], [33]. This localized approach is most appropriate to identify responses directly regulated by nematodes, while studies using systemic tissues can provide a global view of the infection response in host plants and offer a broader perspective on plant health in the context of plant-nematode interactions.

Species within the genus *Hirschmanniella* infest 58% of the world’s rice fields, causing 25% yield losses [1]. *Hirschmanniella oryzae* is one of the few plant-parasitic nematodes that can survive in anoxic environments [34] and hence this nematode is the most common plant-parasitic nematode in rice grown under constantly flooded conditions [1]. In previous studies performed by our research group we investigated (1) the systemic transcriptional responses of selected marker genes involved in hormone related defense pathways using qRT-PCR [10] and (2) the local reaction of rice roots upon *H. oryzae* and *Meloidogyne graminicola* infection using mRNA-Seq [4], [25].

In the current study, a more general overview of the systemic responses of *H. oryzae*-infested rice plants is provided. Using mRNA-Seq we have acquired almost 100 million reads from infected and healthy rice shoot tissues (>5 M per sample), thereby obtaining count data for more than 80,000 annotated and unannotated rice transcripts. We have used a synchronized inoculation method, in which the nematodes only had a limited period (24 hours) to infect roots. This allowed a relatively uniform infection process, so that the transcript changes detected here are not compromised by continuous entry of juveniles.

### Systemic effects on defense responses

Locally, roots infected by *H. oryzae* exhibit a fast (3 dpi) and strong induction of genes involved in cell death as well as defense response pathways, like for instance the phenylpropanoid and jasmonate pathways [4]. The systemic shoot tissues also showed an induction of some defense-related genes at 3 dpi, including genes involved in jasmonate and ethylene production [10] and genes involved in flavonoid and phytoalexin biosynthesis (this study). However, at 7 dpi the expression of many of these genes were significantly downregulated or had returned to non-infected tissue level [10]. In contrast, shoot tissue of Arabidopsis plants infected with the cyst nematode *Heterodera schachtii* contained continuously higher levels of SA and JA-marker genes than corresponding control plants, from 5 till 14 dpi [8]. However, in the case of root knot nematode infection, a strong and early suppression of SA, JA, ET- biosynthesis genes, as well as PR genes has been observed already at 3 dpi [4] and 5 dpi [8], in systemic tissue of the *Meloidogyne graminicola*-rice [4] and *Meloidogyne incognita*-Arabidopsis [8] interaction, respectively. Hence, the here-observed systemic suppression of the plant defense system upon *H. oryzae* infection seems to be slower and less consistent than what was seen in systemic shoot tissues upon root knot nematode infection in rice and Arabidopsis [8].

In the current mRNA-Seq data we found two notable exceptions to this rule, since we detected a repression of the isoprenoid and shikimate pathway already at 3 dpi in the systemic tissue. The suppression of genes from the shikimate pathway could potentially deplete the pool of chorismate and prephenate available for the production of aromatic amino acids and salicylate. Aromatic amino acids are precursors for several secondary metabolites with proven roles in plant defense, like lignin, phenolics, flavonoids and salicylic acid, produced through the phenylpropanoid pathway [35]. Interestingly, we have recently discovered two candidate effectors of *H. oryzae* with homology to chorismate mutase and isochorismatase [36]. These effectors are most likely involved in the deregulation of the shikimate and subsequent phenylpropanoid pathway in the host plant. A secreted chorismate mutase from *Ustilago maydis* has for instance been demonstrated to lower the total salicylic acid content in infected leaves [37]. If and how these effectors can also affect the plant defence system in the above-ground tissue is an intriguing route for further research.

The isoprenoid pathway, which is also suppressed at 3 dpi in systemic shoots, is responsible for the production of a variety of both primary and secondary metabolites with very diverse functions, like tocopherol, carotenoids, mevalonate and provides precursors for brassinosteroid, gibberellin and chlorophyll biosynthesis [38].

Consistent with this view, genes involved in both chlorophyll biosynthesis as well as brassinosteroid biosynthesis and signalling were impaired in the systemic plant tissue. Brassinosteroids (BRs) are polyhydroxysteroids that offer a wide range of physiological activities starting from seed germination till flowering and senescence [39]. The strong suppression of this pathway as seen in the infected plants can potentially have dramatic effects on plant development. Although we did not observe significant effects on plant growth at 3 and 7 dpi, effects at later time points can certainly be expected.

In addition to their well-known role in plant development, BRs have recently been demonstrated to also play a role in plant responses to a broad spectrum of environmental stresses [40], [41]. For instance, BRI1, which was here found to be strongly repressed in the shoots of *H. oryzae* infected plants, is an important player in PAMP triggered Immunity [42].

### Systemic effects on primary metabolism

The data provided in the current study reveals a remarkable suppression of the whole basic metabolic machinery in the rice shoot. The transcriptome revealed changes in the pathways needed for production of different metabolites for plant growth and development, such as carbohydrates, lipids, proteins, and nucleic acids. Similarly, Hofmann et al. [9] showed that cyst nematode (*Heterodera schachtii*) infection was causing dramatic changes in metabolite profiles in systemic root and shoot tissues. For instance, amino acid levels decreased in shoots of cyst-nematode infected plants in comparison with control shoots, but levels of some organic acids were increased [9]. It is known that defense metabolites such as phenylpropanoid pathway products, PR-proteins, ROS and callose represent a major flow of carbon from primary metabolism into secondary metabolism [43]. Large carbon fluxes into secondary metabolism during the defense response cannot occur without influencing reactions in primary metabolism [43]. The local strong defense induction inflicted in the *H. oryzae* infected root system [10], might be responsible for a depletion of carbon and energy available for the primary metabolism of the plant shoot.

### Potential consequences for interactions with other pathogens and herbivores

In the case of root feeding by insects, a general induction of defense responses in the systemic shoot tissue has been observed [12], [44], [45]. For instance in cotton (*Gossypium herbaceum*), root-chewing by insect larvae led to increased concentrations of non-volatile terpenoid compounds in the leaves, known to deter insect feeding [12]. Recently Wondafrash et al. [15] provided a review of the systemic effects that root feeding by nematodes can have on subsequent attack by above-ground herbivores. They concluded that the effects varied depending on the feeding style of the nematode, nematode population densities and the susceptibility of the host plant, and different outcomes have been reported. For instance, soybeans with moderate and high *Heterodera glycines* infection levels supported greater *Helicoverpa zea* larval populations than observed on control or low density *H. glycines* infested plants. Nevertheless, very high *H. glycines* population densities caused severe stunting and chlorosis, reducing the suitability of these soybeans as a food source for *H. zea* larvae [46]. However, in Arabidopsis, a simultaneous inoculation with the sedentary endoparasitic nematode *H. schachtii* and the aphid *Brevicoryne brassicae* was performed [47] and no effects of the nematode infection were observed on the aphid performance at day 3. Again different, *Agrostis capillaris* and *Anthoxanthum odoratum* plants attacked by ectoparasitic and migratory endoparasitic nematodes, caused a reduced fecundity of the aphids *Rhopalosiphum padi* feeding on the shoots [48]. A lower amino acid content was observed in the phloem sap of the nematode-infected plants, and was probably one of the causes of this decreased aphid performance. Since our transcriptome data provides evidence for a reduced amino acid production in the above-ground tissues of *H. oryzae* infected rice plants, one could deduce that this tissue might be less attractive to phloem-feeding insects. Nevertheless, experimental evidence for this is currently lacking.

In conclusion, this transcriptome analysis of above-ground tissues upon rice root nematode infection unveils a major impairment of primary metabolite biosynthetic pathways in the shoots of infested plants. Biochemical investigation of chlorophyll levels in these tissues, corroborated the significant reduction of the plant’s photosynthetic capacity upon *H. oryzae* infection. Although less prominent, some subtle changes in defence-related pathways were also detected, among which mainly the isoprenoid and shikimate pathway seem to be negatively affected. If and how these changes affect the abiotic and biotic stress tolerance of the surviving rice plants remains to be further investigated.

## Supporting Information

Figure S1 **Mapman visualization of the expression profiles of genes involved in the general metabolism of the rice plant.** The visualization shows the observed differential expression patterns, based on the Log_2_ fold changes of mRNA levels, in shoots of infected versus uninfected control plants at 3 dpi (A) and 7 dpi (B). Red dots indicate that the gene is upregulated in infected plants versus the corresponding healthy control plants, while blue indicates downregulation.
(ZIP)

Table S1 **Differentially expressed genes (FDR<0.1), when comparing systemic tissue of** ***H. oryzae*** **infected plants with uninfected plants at time point 3 dpi.** The table shows the rice locus number, the annotation (MSU7.0), the Log2 of the fold-change (FC) of infected versus uninfected shoot tissue at 3 dpi and the FDR-value. Novel transcriptionally active regions received a gene ID starting with RNSR. FDR: false discovery rate. RNSR: Root Nematode Systemic Respons.
(XLSX)

Table S2 **Differentially expressed genes (FDR<0.1), when comparing systemic tissue of** ***H. oryzae*** **infected plants with uninfected plants at time point 7 dpi.** The table shows the rice locus number, the annotation (MSU7.0), the Log2 of the fold-change (FC) of infected versus uninfected shoot tissue at 7 dpi and the FDR-value. Novel transcriptionally active regions received a gene ID starting with RNSR. FDR: false discovery rate. RNSR: Root Nematode Systemic Respons.
(XLSX)

Table S3 **List of putative novel transcriptionally active regions identified in the investigated rice shoot material, with their genomic location, BLASTn results against cDNA and genomic DNA of** ***Oryza sativa*** **cv. ‘Japonica’, BLASTx results against rice proteins, SwissProt/tremble.** For all analyses a bitscore cut-off of 50 was used.
(XLSX)
